# The Impact of Controlled Physical Training with Hydrotherapy on Changes in Swelling and Claudication Distance in Patients with Atherosclerotic Ischemia of the Lower Limbs

**DOI:** 10.3390/ijerph192315715

**Published:** 2022-11-25

**Authors:** Joanna Kapusta, Robert Irzmański

**Affiliations:** Department of Internal Diseases, Rehabilitation and Physical Medicine, Medical University of Lodz, 70-445 Lodz, Poland

**Keywords:** physical activity, controlled training, hydrotherapy, cardiac rehabilitation, atherosclerosis, peripheral circulation disorders

## Abstract

Background: Patients with peripheral artery disease (PAD) often experience intermittent claudication. It is manifested by pain typically seen in the distal part of the legs during walking, which impairs the ability to walk, limits physical activity and results in lower health-related quality of life. It often leads to chronic ischemic pain, ulceration and even amputation. The aim of the study was to evaluate the effect of controlled physical training and whirlpool massage on changes in circuits, range of motion and distance of claudication in people with atherosclerotic ischemia of the lower limbs. Material and methods: The study included 100 patients, males and females aged 39 to 79 years, with peripheral circulation disorders of the lower limbs. The subjects were randomly divided into two groups. Group I (G) was treated with a series of 10 lower-limb whirling massages and took part in individually planned training, including breathing, relaxation and active free lower-limb exercises. Group II-control group (GC) took part only in individually planned training. Prior to the treatment procedures and after their completion, the ranges of movement of the ankle joint and the circumference of the lower limbs were measured and the corridor test (6MWT, six-minute walk test) was performed. Results: Statistically significant reduction in the circumference of the foot, ankle, calf and thigh in the G group was noticed. Both, in G and in GC group, a statistically significant increase in the range of dorsiflexion of the foot was found in comparison to the period before the procedures (*p* = 0.010; *p* = 0.006, respectively). There was also a statistically significant increase in the range of motion of the plantar flexion of the foot after the procedures in G (*p* = 0.007) and in GC (*p* = 0.048). Differences in the circumference of the lower limbs and the range of ankle joint movements between the study group and the control group before and after the therapy were not statistically significant. However, there was a statistically significant difference between these groups after the procedures in the 6-min walk test—significantly higher values of 6MWT were recorded in group G (*p* = 0.003). Conclusions: Individually planned training, supplemented with hydrotherapy as thermal therapy, has a beneficial effect on reducing swelling of the lower limbs, increasing the range of foot movements and extending the distance in the 6-min walk test.

## 1. Introduction

Peripheral arterial disease (PAD) may present different clinical manifestations depending on the location of pathological changes. It may affect the carotid and vertebral arteries, as well as arteries of the upper and lower limbs. Regardless of the affected region, the underlying cause of PAD is always atherosclerosis. The occurrence of coronary artery atherosclerosis adds to the overall cardiovascular risk. Thus, besides treating the symptoms, in the management of patients with PAD, a special focus should be placed on the diagnosis and modification of risk factors [1].

Atherosclerotic ischemia of the lower limbs may produce a range of symptoms depending on the location of lesions, disease progression, degree of vessel occlusion and length of the affected vessel segment.

One of the symptoms of lower limb ischemia reported by the patients, but often ignored, is a sensation of cold, or coolness, either in the toes or the entire foot. The manifestation most readily associated with PAD is intermittent claudication. The increased demand for oxygen upon exertion coupled with insufficient supply resulting from partial or complete atherosclerotic occlusion of an artery may lead to pain, usually in the distal segments of the lower limbs, which subsides following several minutes of rest. During exercise, patients with PAD typically complain of strong cramps or calf fatigue, although the symptoms may also appear in the thighs and buttocks. Sometimes intermittent claudication symptoms may be difficult to observe due to the patient’s low physical activity or the presence of a comorbidity, such as a severe lung disease or musculoskeletal condition impairing mobility [2].

In late-stage PAD, when tissue perfusion is no longer able to meet the metabolic demands of the tissues, critical limb ischemia can develop. This often leads to chronic ischemic pain, ulceration and eventually even amputation [3,4]. Patients are recommended to perform physical activity (i.e., walks lasting at least 30–45 min, walks 3 times a week) [3,5,6]. It is also recommended practice to participate in walking training sessions on a treadmill, under the supervision of qualified medical staff [3,7]. Training should last about 35 min and consist of several cycles in the exercise-rest pattern. However, walking training is not recommended in patients with severe ischemic changes (class IV, according to the Fontaine scale). Moreover, one should remember about the contraindications to exercise, i.e., diseases of the musculoskeletal system, diseases of the nervous system and severe coronary artery disease [3,7,8]. Since this is a serious problem, effective treatments for PAD patients are still being sought. Promising results were observed with the use of correlated therapies using thermal stimuli, massage and exercise [9,10,11,12]. Structural changes related to the thermal stimulus, haemodynamic and vascular changes may lead to favorable adaptive changes in the skin microcirculation and thus play a key role in the prevention of ischemic ulcers. However, the mechanisms underlying the hemodynamic and structural adaptations induced by heat therapy are poorly understood [4].

Therefore, the aim of this study was to evaluate the effect of controlled physical training together with whirlpool massages on changes in circuits, range of motion and claudication distance in people with atherosclerotic ischemia of the lower limbs.

## 2. Materials and Methods

The study encompassed 100 male and female patients aged 39 to 79 years with peripheral arterial disease of the lower limbs (Fontaine stages I and II). Degrees of lower limb ischemia according to Fontaine’s classification are presented in Table 1. The participants were divided into two equinumerous groups. The study group (G) consisted of 21 females and 29 males, with a mean age of 59.7 ± 11.7 years. The control group (GC) was comprised of 20 females and 30 males, with a mean age of 60.3 ± 11.6 years.

All participants in both groups took medications prescribed pursuant to the standards of the Polish Cardiological Society [13]. Furthermore, the two groups were comparable in terms of mean systolic and diastolic pressure.

The criteria for patient inclusion in the study were as follows:

1. a peripheral circulation disorder affecting the lower limbs (due to pathological changes in the peripheral vessels of the lower limbs), confirmed by Doppler ultrasound examination;
2. informed consent of the patient;
3. nonparticipation in other physical therapy treatments;
4. absence of contraindications for participation in the study, such as acute inflammatory conditions, fever, arterial hypertension (systolic pressure > 200 mmHg or diastolic pressure > 100 mmHg), respiratory or circulatory insufficiency, Fontaine stage III or IV PAD of the lower limbs, infectious diseases, pyogenic skin lesions, existing or imminent thrombosis and varicose veins of the lower limbs.

Patients who did not meet even a single criterion were not qualified for the study program.

Before participating, the patients were informed about the objectives of the study and how it was conducted and then gave their informed consent to participate in the study. The study was conducted in accordance with the guidelines of the Helsinki Declaration and approved by the Bioethics Committee of the Medical University of Lodz (approval number RNN/380/14/KB).

### 2.1. Controlled Physical Training Program

Following preliminary qualification for the study, the participants were divided into two groups. The members of the study group were administered 10 whirlpool treatments of the lower limbs and participated in an individually prescribed training program, while the controls participated only in a training program designed in the same way as for the study group. Patients participated in hydrotherapy and physical exercises from Monday to Saturday. The total duration of therapy was 21 days (3 weeks).

Exercise program: Exercises were performed under the supervision of a physiotherapist, every 2 days, for 30 min., and included breathing exercises, relaxation exercises and active free lower limbs, such as toe climbing, dorsal and plantar flexions. During the exercises, the Borg scale did not exceed 11 points.

Physical exercises were performed 10 min after the end of the hydrotherapy treatment (this was the time for the patient to wipe off the water, get dressed and go to the kinesitherapy room.)

A whirlpool bath for the lower limbs was performed in special tubs adapted to the lower limbs. During the procedure, the patient was in a sitting position and immersed his lower limbs in the water so that the nozzle causing the swirling motion of the water was at the level of the patient’s lower leg. The water pressure during the treatment was 2.5–3.5 kPa. The treatment was performed every other day, in the afternoon, in water at a temperature of about 39 °C (39.3 ± 0.5 °C) for 20 min. Hydrotherapy treatments were performed using a tub for whirling hydromassage of the lower limbs, in the foot and lower leg zones—Aquanesis P. The device has a thermometer with a water temperature sensor, thanks to which a constant water temperature was maintained throughout the treatment.

### 2.2. Evaluation of the Range of Motion in the Talocrural Joint

Before and after conducting the prescribed series of training sessions with or without hydrotherapy treatments, all participants had the ROM in the talocrural joints measured in degrees [°] upon active flexion (plantarflexion) and active extension (dorsiflexion).

During the measurement of active plantarflexion, the patient was placed in the prone position with the feet hanging freely off the examination table so that the talocrural joint was in the intermediate position, that is, the long axes of the foot and shin were at right angles. The goniometer was placed over the lateral aspect of the lateral malleolus, with the stationary arm of the goniometer aligned along the lateral midline of the lower leg, pointing towards the head of the fibula, and with the moving arm placed along the fifth metatarsal (the lateral edge of the foot). The patient was asked to extend the forefoot on their own.

During the measurement of active dorsiflexion, the patient was in the sitting position with the lower legs hanging freely so that the talocrural joint was in the intermediate position. The goniometer was placed over the lateral malleolus, with the stationary arm of the goniometer aligned along the lateral midline of the lower leg and with the moving arm placed along the fifth metatarsal, parallel to the floor. The patient was asked to bring the forefoot up on their own.

Table 2 shows estimated normal ROM values for active plantarflexion and dorsiflexion in the talocrural joint.

According to the sagittal, frontal, transverse, rotation (SFTR) system, the estimated normal ROM values for active plantarflexion and active dorsiflexion at the talocrural joint are as follows (Table 3):

### 2.3. Lower Limb Circumference Measurements

Lower limb circumference measurements [cm] were taken before and after conducting the prescribed series of training sessions and/or hydrotherapy treatments. The patients were placed in the supine position. Measurements were made on one lower limb and included forefoot bulk measured at the level of the heads of the first and fifth metatarsals; calf 2-ankle circumference taken immediately above the ankle bones; calf 1- calf circumference taken 15 cm below the apex of the patella; and thigh 2 and 1-thigh circumference taken 10 cm and 15 cm above the base of the patella.

### 2.4. Six-Minute Walk Test

The six-minute walk test (6MWT) assesses the distance that a patient is able to cover over six minutes on a flat, hard surface. The test is used to determine submaximal aerobic capacity and exercise tolerance in individuals with circulatory and respiratory disorders; it may also be used to evaluate the levels of motor control and muscle metabolism. The test can be conducted both indoors and outdoors.

In the present study, the 6MWT was conducted pursuant to the guidelines of the American Thoracic Society [15]. The walkway was 30 m long with intermediate points marked every 3 m with cones; the turnaround points at either end of the walkway were designated with chairs. Prior to the test, the patients were told that the objective was to walk as far as possible for 6 min, that they should walk at their own pace (but not run or jog) and that they were permitted to stop and rest at any time during the test if necessary. Prior to the test, the patients rested in the sitting position for 10 min. Resting pulse was taken, and blood pressure was determined with a medical sphygmomanometer. Time was measured with a stopwatch. After the test, the distance walked over 6 min was recorded, and pulse and pressure were taken again. The patients rated perceived fatigue levels on the Borg scale, which is an instrument designed for self-assessment of exercise fatigue.

### 2.5. Statistical Analysis

Calculations were made using the STATISTICA PL v. 7.1 (StatSoft, Poland) and PQSTAT 1.6.4 (PQStat Software, Poland) statistical packages and the WRS2 v.0.9-2 package (Mair & Wilcox (2017) Mair P, Wilcox R. WRS2: a collection of robust statistical methods (Version: 0.9-2) 2017.) in the R v. 3.4.2 environment. Quantitative variables were characterized by basic descriptive statistics: mean, median, lowest and highest value, quartiles-first and third (Q1 and Q3) and standard deviation (SD). In the case of quantitative variables, the hypothesis about the normality of distributions was verified using the Shapiro–Wilk test of normality. The Student’s t test or the Mann–Whitney U test (depending on the normality of distribution of the examined variables) were used to compare the control group and the study group in terms of age, weight, height and BMI. To compare the groups (between each other and over time), the “robust” analysis of variance for repeated measures (package WRS2) was used due to the lack of normality of distributions. The Yuen test was used for pairwise comparisons. Sidak’s correction was taken into account in multiple comparisons. The strength of the relationship between the variables was determined by calculating Spearman’s correlation coefficients. Results at *p* < 0.05 were considered statistically significant.

## 3. Results

### 3.1. Evaluation of Basic Characteristics

The study group consisted of 21 females and 29 males (42% vs. 58%), and the control group consisted of 20 females and 30 males (40% vs. 60%). The two groups did not differ significantly in terms of gender composition (*p* = 0.839).

The mean age of patients was 59.7 ± 11.7 years in the study group and 60.2 ± 11.6 years in the control group, while the respective mean body mass index (BMI) values were 29.9 ± 4.7 kg/m^2^ and 30.0 ± 3.4 kg/m^2^ (Table 4). No statistically significant differences in terms of age or BMI were found between the groups.

### 3.2. Analysis of Changes in Lower Limb Circumferences

**Figure 1:**
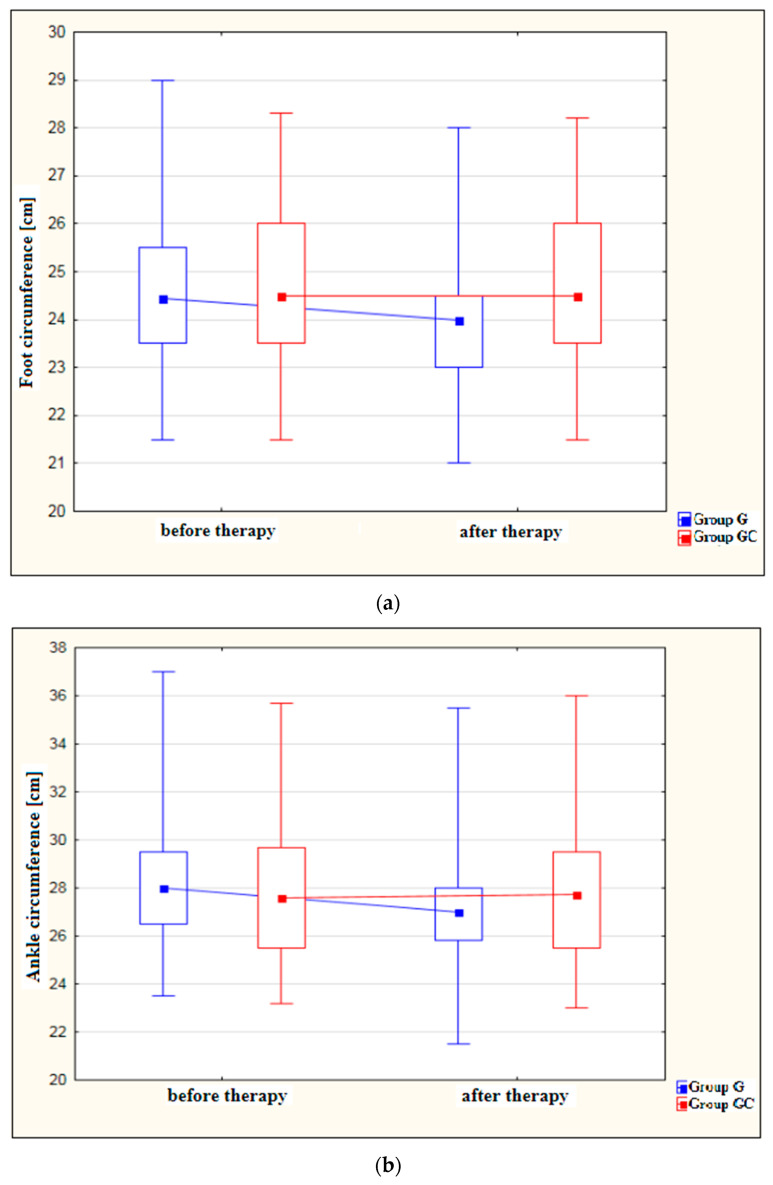

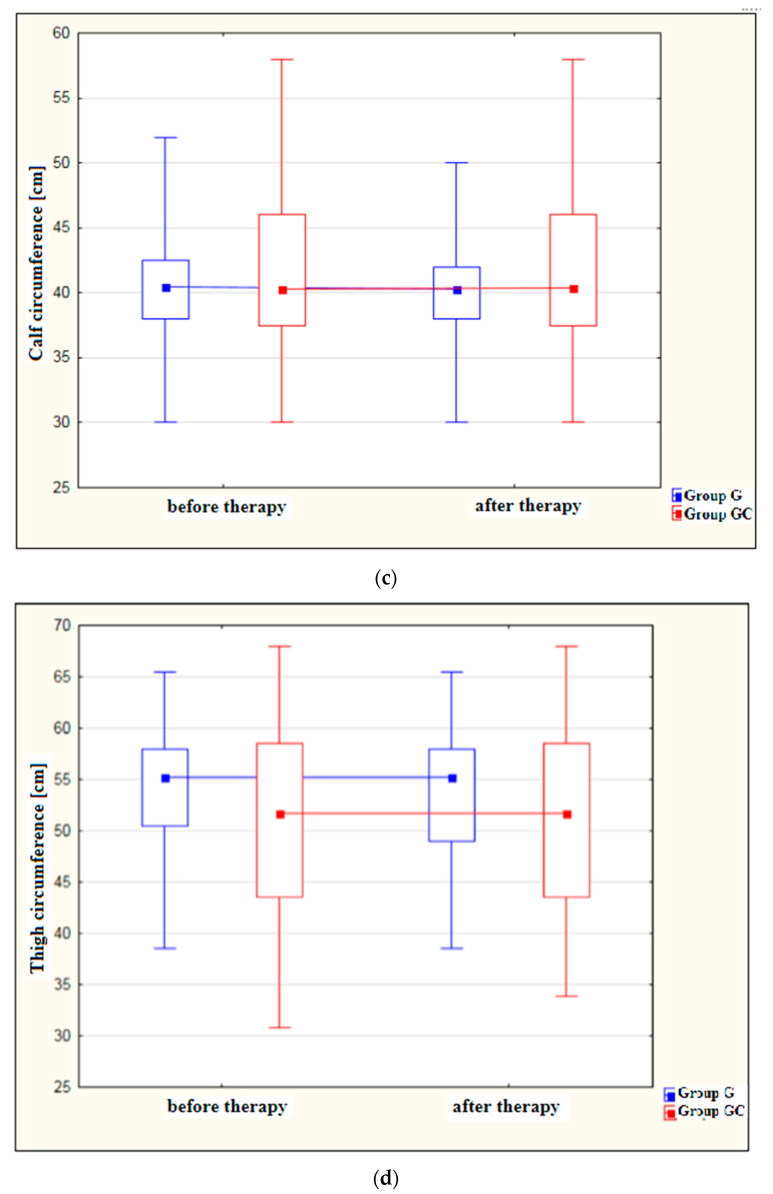
(**a**) Changes in foot circumference [cm] in the study and control groups, before and after therapy. (**b**) Changes in ankle circumference [cm] in the study and control groups, before and after therapy. (**c**) Changes in calf circumference [cm] in the study and control groups, before and after therapy. (**d**) Changes in thigh circumference [cm] in the study and control groups, before and after therapy.

Figure 1a–d shows results for foot, ankle, calf and thigh circumferences [cm] in the study and control groups before and after the administered course of treatments.

A statistically significant decrease in foot circumference after therapy was found in the study group (*p* = 0.029). The difference in foot circumference after the procedures in the control group (GC) was not statistically significant (*p* = 1.000).

Also, the ankle circumference was significantly lower post-therapy in the study group at (*p* < 0.001). The difference in ankle circumference before and after the procedures in the control group (GC) was not statistically significant (*p* = 1.000).

Calf circumference was found to decrease significantly after treatment in the study group (*p* = 0.024). The difference in calf circumference before and after the procedures in the GC group was not statistically significant (*p* = 1.000).

Measurements revealed a significant reduction in thigh circumference following therapy in the study group (*p* = 0.020). Again, the difference before and after therapy for the GC group failed to reach statistical significance (*p* = 1.000).

### 3.3. Analysis of Changes in the ROM of the Talocrural Joint

**Figure 2:**
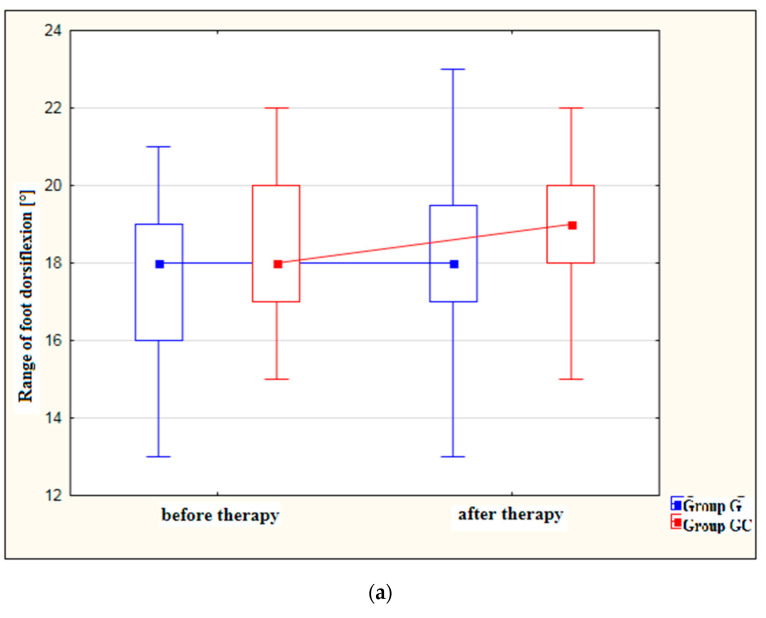

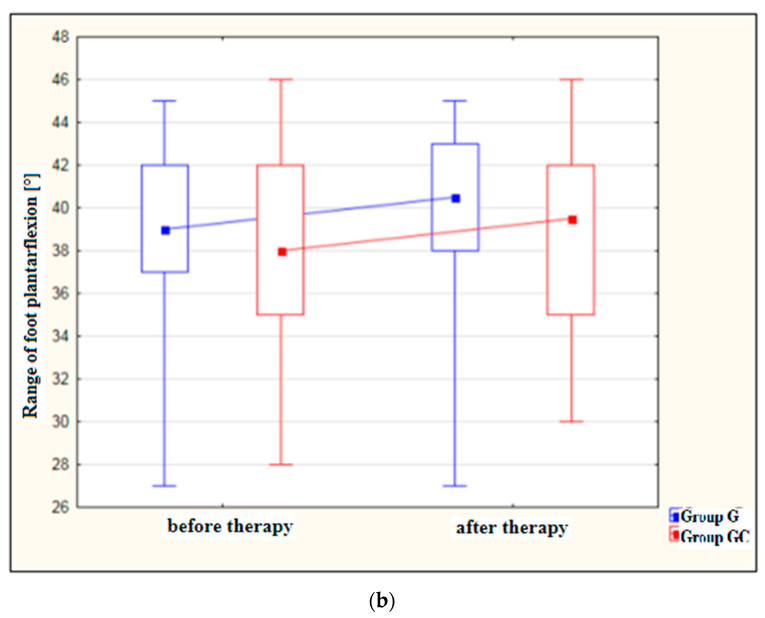
(**a**) Changes in the range of foot dorsiflexion [°] in the study and control groups, before and after therapy. (**b**) Changes in the range of foot plantarflexion [°] in the study and control groups, before and after therapy.

Figure 2a,b presents results for foot dorsiflexion and plantarflexion [°] in the study and control groups before and after therapy.

After treatment, both the study and control groups revealed a statistically significant improvement in the range of dorsiflexion (*p* = 0.010 and *p* = 0.006, respectively) as well as in the range of plantarflexion (*p* = 0.007 and *p* = 0.048, respectively). However, greater improvement was noted in the group with the additional thermal stimulus.

### 3.4. Analysis of Changes in 6MWT Performance

**Figure 3:**
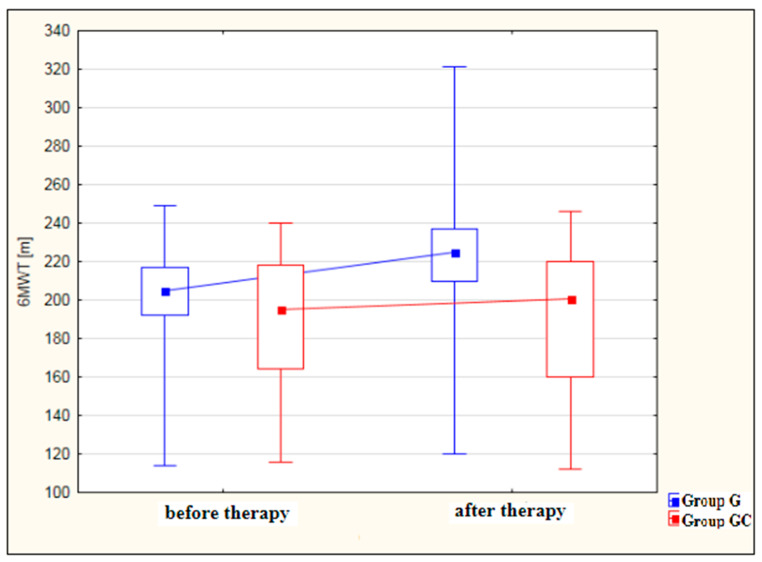
Changes in 6MWT distance [m] in the study and control groups, before and after therapy.

Figure 3 shows an analysis of 6MWT distance results [m] for the study and control groups before and after therapy. The improvement in 6MWT performance after therapy was statistically significant for the study group (*p* < 0.001) but not for the GC group (*p* = 0.980). While, before therapy, the results obtained by the study and control groups did not differ significantly, after therapy, the performance of the study group was significantly better (*p* = 0.003) than that of the controls.

In Table 5, the results of the tested parameters (lower limb circumference, range of motion and six-minute walk test) are presented as a comparison between the study groups (G vs. GC).

Differences in foot, ankle, calf and thigh circumference, between the control and study groups, before and after therapy were not significant. Differences in the range of foot dorsiflexion and plantarflexion, between the control and study groups, before and after therapy were also not significant.

In 6MWT, no statistically significant difference between the groups (G vs. GC) was observed before the treatments. However, there is a statistically significant difference between these groups after the treatments—significantly higher values of 6MWT were recorded in group G (*p* = 0.003).

### 3.5. Correlations between 6MWT Distance and Lower Limb Circumferences

**Figure 4:**
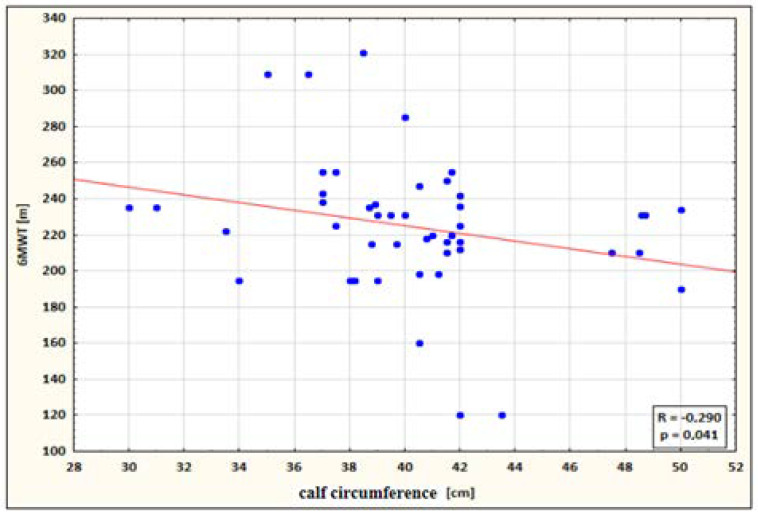
Correlation between calf circumference and 6MWT performance for the study group after therapy.

**Figure 5:**
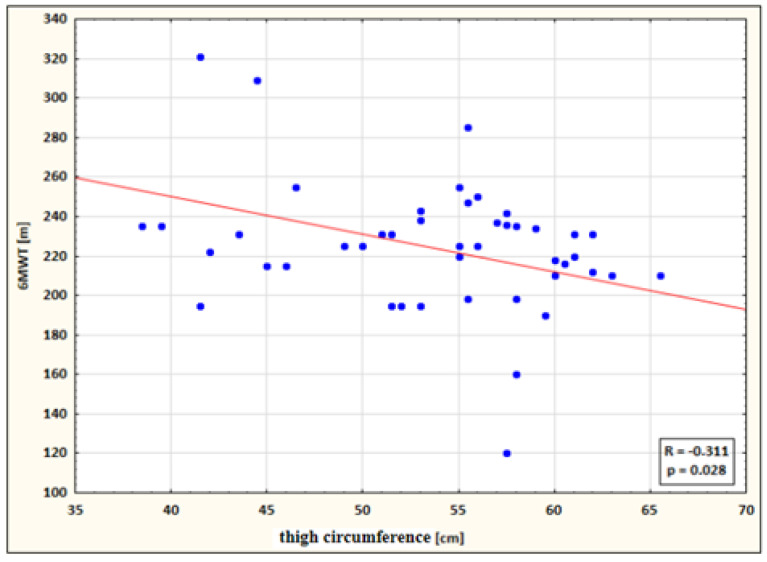
Correlation between thigh circumference and 6MWT performance for the study group after therapy.

Significant correlations in the study group after therapy (Table 6):

The following statistically significant correlations were identified:

- a small negative correlation between calf circumference and 6MWT performance for the study group after therapy (the higher the circumference, the shorter the 6MWT distance) (Figure 4);

- a small negative correlation between thigh circumference and 6MWT performance for the study group after therapy (the higher the circumference, the shorter the 6MWT distance) (Figure 5).

## 4. Discussion

Atherosclerotic ischemia of the lower limbs may present different clinical manifestations depending on the location of pathological changes, disease progression, the degree of vessel occlusion and the length of the affected vessel segment. The symptoms of lower limb ischemia include limb edema, which significantly decreases the patients’ quality of life. A comprehensive etiological classification of edemas was presented by Olszewski in 1991 [16]. Accordingly, primary edema is caused by lymphatic system anomalies, while secondary edema may arise due to a variety of disorders, including chronic venous insufficiency, trauma, infections and superficial and/or deep vein thrombosis [17]. In turn, the underlying factors of vascular edema are usually incompetent or hypoplastic venous valves. Edema is typically located in the distal parts of limbs but may affect not only the foot and talocrural joint but also the calf, knee joint and thigh [18]. As many as 30% of cases of edema are attributable to deep vein thrombosis, which is itself caused by so-called Virchow’s triad, consisting of vessel wall injury, hypercoagulability and stasis of blood flow [17]. Edema and its consequences, such as reduced ROM in the lower limb joints, significantly impair the quality of life in patients with PAD. Fiodorenko-Dumas et al. [19] studied the effects of edema associated with vascular diseases on lower limb circumferences, the range of motion in lower limb joints and the quality of life of the patients. The study involved 30 individuals suffering from idiopathic lymphatic insufficiency, chronic venous insufficiency-related swelling and swelling caused by atherosclerosis and thrombosis. The ROM of the lower limbs was determined using a goniometer according to the ISOM standards, while the circumferences (ankle, calf 1 and 2, thigh 1 and 2) were taken with a tape measure. The quality of life was evaluated by means of an originally developed questionnaire and the visual analog scale (VAS scale). The results indicated a significantly compromised quality of life correlated with edema in the talocrural joint, which resulted in substantially impaired mobility, especially in terms of foot dorsiflexion, supination and pronation.

The present work included analysis of foot, ankle, calf and thigh circumferences before and after therapy. Only G group, which was administered a series of whirlpool treatments, revealed a statistically significant decrease in all of these parameters after therapy. Differences in circumferences, between G and GC groups before and after therapy, were not significant. In terms of dorsiflexion and plantarflexion, significant post-therapy improvement was recorded for both the study group and for the control group. Whereas, differences in the ankle range of movement, between the groups, before and after therapy were not significant. Furthermore, in both groups, a small negative correlation was found between the range of motion and some of the lower limb circumferences. The obtained results show that lower limb edema caused by vascular conditions significantly affects the ROM in the talocrural joint, impairing the gait physiology of patients and thus lowering their quality of life.

A substantial body of research has shown that chronic lower limb ischemia leads to considerable impairment of patients’ daily activity. Management standards in clinical practice are focused on individualized approach to patients and their involvement in designing therapy programs. Following these guidelines, health care professionals should conduct a multi-faceted evaluation of the patient’s health. Unsurprisingly, healthy individuals exhibit higher life quality indicators as compared to patients with PAD [20]. However, noninvasive management of PAD has been found to improve claudication distance and the quality of life. Health-related quality of life evaluation is of fundamental importance, taking into account the interrelationships between the severity of symptoms and the physical, social and emotional condition of the patients [20].

Individually prescribed programs of home-based exercises are the preferred form of training for patients with chronic ischemia of the lower limbs. The pace of the exercise and the number of repetitions should be adjusted to the physical capacity of each patient, taking into consideration their age, mobility impairments and pain complaints. If supervised training in a specialized institution is impossible, home-based programs are an important therapeutic alternative for patients with PAD [21]. Systematic physical exercises have a beneficial effect on the patient’s walking capacity; for instance, in a study by Degischer et al. [5] and McDermott et al. [22], supervised physical training in patients with PAD improved their 6MWT performance and functional capacities.

In our study, in 6MWT, statistically significant difference between the groups (G vs. GC) was observed after the treatments—significantly higher values of 6MWT were recorded in group G (*p* = 0.003).

Meta-analysis of randomized controlled trials show that supervised exercise improves more than regular healthcare, maximum walking distance (average 120 m) and pain-free distance (average 80 m) in patients with PAD [23,24,25]. Unfortunately, in this group of patients, the performance of prolonged and vigorous exercise is limited due to chronic ischemic pain associated with peripheral circulatory disturbance [26,27].

Parszakow et al. [28] investigated the reactivity of cutaneous microcirculation in a group of patients with PAD. They assessed the temperature change on the sole of the big toe in response to local heating. The researchers found that in people with PAD, the activity of the endothelium, smooth muscles and the nervous system during heating was significantly lower compared to healthy controls [4]. Heat therapy, which is the use of an external heat source to raise local or systemic body temperature, has proven to be an effective intervention in patients with PAD [29]. The use of a thermal stimulus has found application in hydrotherapy. This therapy covers a wide range of methods, such as immersion in specially adapted bathtubs or external heating using water-circulating cuffs for the lower limbs. During vigorous exercise, when tissue temperature rises, cardiac output increases and peripheral resistance decreases [30], while local heating causes vasodilation and reduction of peripheral vascular resistance [31]. Both exercise and heat therapy have similar effects in terms of hemodynamics [9,10], but unlike exercise itself, the reaction to heat does not cause a quick feeling of fatigue [27]; therefore, the combination of these two therapies seems justified.

Akerman et al. studied the differences in basic cardiovascular hemodynamics in patients with peripheral arterial disease in response to a 12-week heat therapy program and a 12-week supervised exercise program [10]. One group received heat therapy, which included bathing in water at a temperature of about 39 °C, 3–5 days/week for ≤30 min as well as ≤30 min of supervised gymnastic exercise. The control group participated only in supervised walking and gym exercises, 1–2 days a week, ≤90 min. The authors observed that after three months in the group with the additional thermal stimulus, better results were obtained in the 6MWT and resting blood pressure tests. Moreover, this form of combination therapy was better tolerated by the patients. A similar study was conducted by Song-Young Park et al. [11] The authors analyzed the effects of 12-week treatments: treadmill training and training including warm water exercises in patients with PAD. Both groups showed significant reductions in blood pressure, resting heart rate and pulse wave velocity and significantly increased 6MWT compared to the baseline, with the effects being much more pronounced in the warm water group [11,12]. Heat therapy safely and effectively generates cardiovascular adaptation mechanisms in patients with PAD; it also reduces pain and improves general physical fitness [10,11,12]. Therefore, the combination of controlled physical training with a thermal stimulus may be a promising form of conservative treatment in the early stage of PAD [32].

This study was aimed at assessing the effect of physical activity in the form of systematic exercises and hydrotherapy treatments as heat therapy on improving the range of motion, reducing swelling and extending the distance of claudication in patients with atherosclerotic ischemia of the lower limbs. The results obtained from our own research authorize us to state the beneficial effect of the procedures performed in this group of patients.

*Strengths and limitations of the study.* Physical activity in the form of exercise, supplemented with hydrotherapy as thermal therapy, increased the patients’ tolerance to exercise. Therefore, this study highlights a new opportunity for PAD patients to obtain cardiovascular and functional benefits from conservative treatment when exercise is limited. The strength of the presented study is that our observations do not concern complex therapy or apparatus; therefore, they can be used in public health care facilities, without huge financial outlays. In addition, heat therapy alone or in combination with supervised exercise can reduce the need for more invasive and costly interventions. This therapeutic approach may also allow the cardiovascular system to improve in order to obtain better results after surgical interventions. Our study, in addition to heat therapy, also focused on controlled physical training. Performing exercises under the supervision of a therapist increases the patient’s mobilization, a sense of security and eliminates the risk of improper exercise, which is very important, especially from the patient’s point of view. Our study, although it confirms the observations of a few authors regarding the beneficial effect of hydrotherapy on changes in walking distance (in 6MWT) in patients with PAD, also presents the results obtained in terms of the following parameters: circumferences of the lower limbs and the range of motion of the ankle joint, which as far as we know, have not been previously analyzed in other studies. However, the presented study had some limitations. The limiting factor is the small size of the subgroups and the relatively short observation period. Therefore, we cannot conclude whether the observed improvement lasted longer. It is possible that further intervention would also have a positive effect on the parameters studied. In addition, this study did not analyze the potential mechanisms of the observed improvement, such as assessment of endothelial function, structural changes by Doppler ultrasound or assessment of skeletal muscle mitochondrial function. Also, the study group was limited to patients with a mild stage of PAD (Fontaine’s stage I/II). Therefore, these results cannot be generalized for patients with severe forms of PAD but may be extended to include patients with more advanced disease in the future. It may also be important to standardize methods in this area to make the results more comparable. Therefore, further studies to confirm the clinical benefits of hydrotherapy and its combination with other treatments will be required.

## 5. Conclusions

Based on the results of our research, it can be concluded that, in patients with atherosclerotic ischemia of the lower limbs, the combination of exercise with hydrotherapy gives more beneficial effects than exercise alone, as evidenced by a significant increase in the total distance in the 6-min walk test. Although the range of motion of the ankle joint increased after the procedures in both groups, there was no significant difference between the exercise-only group and the hydrotherapy and exercise group. In terms of lower limb circumferences-only, the combined therapy group had clinically significant results after the treatments. Therefore, supplementing physical training with a thermal stimulus is a promising form of conservative treatment in patients with atherosclerotic ischemia of the lower limbs.

## Figures and Tables

**Table 1:** Degrees of lower limb ischemia according to Fontaine’s classification [3].

Stage of Advancement	Clinical Symptoms of Lower Limb Ischemia
I	no clinical symptoms or the presence of tingling and increased sensitivity of the feet to cold
IIa	intermittent claudication distance > 200 m
IIb	intermittent claudication distance < 200 m
III	pain in the limbs at rest
IV	ulceration, gangrene of the limbs

**Table 2:** Estimated normal active ROM values for the talocrural joint in different age groups [14].

Range	Age [Lat]	Active Plantar Flexion [°]	Active Dorsiflexion [°]
I	18–40	45°	20°
II	41–60	40°–45°	15°–20°
III	61–85	35°–40°	15°

**Table 3:** Estimated normal values for active plantarflexion and dorsiflexion in the talocrural joint [14].

Age Group	Plane of the Study	Standards of the Range of Movements of Active Flexion and Extension
I	S	20–0–45
II	S	(15–20)–0–(40–45)
III	S	15–0–(35–40)

S—sagital plane.

**Table 4:** Characteristics and comparison of the study and control groups in terms of age [years] and BMI [kg/m^2^].

Parameter/Volume	G	GC
Age		
Mean ± SD	59.68 ± 11.74	60.28 ± 11.65
Median Me (IQR)	62 (51–70)	62.5 (52–70)
BMI [kg/m^2^]		
Mean ± SD	29.91 ± 4.76	30.06 ± 3.40
Median Me (IQR)	29.76 (27.34–31.96)	29.64 (27.64–32.53)

BMI—Body Mass Index; SD—standard deviation.

**Table 5:** Comparison of the studied parameters between the study group and the control group.

Variable	G vs. GC before Therapy	G vs. GC after Therapy
Foot circumference	*p* = 0.937	*p* = 0.161
Ankle circumference	*p* = 0.982	*p* = 0.975
Calf circumference	*p* = 0.995	*p* = 0.961
Thigh circumference	*p* = 0.336	*p* = 0.411
Range of foot dorsiflexion	*p* = 0.308	*p* = 0.612
Range of foot plantarflexion	*p* = 0.526	*p* = 0.207
6MWT	*p* = 0.494	*p* = 0.003

G—study group; GC—control group; 6MWT—six-minute walk test.

**Table 6:** Significant correlations between 6MWT distance and lower limb circumferences in the study group after therapy.

Variables	Study Group	
	After	
	R	p
6MWT & Calf Circumference P	−0.290	0.041
6MWT & Thigh Circumference P	−0.311	0.028

## Data Availability

The data underlying this article cannot be shared publicly for the privacy of individuals that participated in the study.
